# Patient specific regulation of metabolism and cell death pathways in m.3243A>G mutated iPS-cells

**DOI:** 10.1038/s41598-026-43696-1

**Published:** 2026-04-27

**Authors:** Sanna Ryytty, Katriina Nurminen, Teemu Tiukuvaara, Petri Mäkinen, Anu Suomalainen, Riikka H. Hämäläinen

**Affiliations:** 1 A. I. Virtanen Institute, University of Eastern Finland, Kuopio, 70210 Finland; 2 Stem Cells and Metabolism Research Program, Research Programs Unit, University of Helsinki, Helsinki, 00290 Finland; 3 HUSLab, Helsinki University Hospital, Helsinki, 00290 Finland

**Keywords:** m.3243A>G, iPSC, apoptosis, BCL2, ferroptosis, Cell biology, Genetics, Molecular biology, Stem cells

## Abstract

The m.3243A>G mitochondrial DNA mutation is one of the most common pathogenic variants associated with mitochondrial disease and its clinical manifestations vary widely among patients. To investigate the mechanisms underlying this variability, we utilized induced pluripotent stem cells (iPSCs) derived from two unrelated patients (P1 and P2) carrying the m.3243A>G mutation. Despite similar heteroplasmy levels, we observed clear patient-specific differences in how the mutations affected iPSC growth and cell death pathways. In patient 2 iPSCs, m.3243A>G reduced cell proliferation and shifted metabolism towards glycolysis, while in patient 1 cells, both oxidative phosphorylation (OXPHOS) and proliferation were slightly enhanced. Susceptibility to apoptosis also differed markedly between the patients. The m.3243A>G mutations reduced apoptosis in P2 iPSCs, in a BCL2 dependent manner, while ferroptosis-related markers such as lipid peroxidation and expression of iron uptake genes were upregulated, suggesting activation of an alternative cell death pathway. Further, ferroptosis inhibition rescued the proliferation defect in P2 iPSCs. In P1, the mutations did not induce cell death by either pathway. Together, these findings demonstrate that the m.3243A>G mutations lead to distinct, patient-specific cellular phenotypes in iPSCs, shaped by differential metabolic reprogramming and cell death regulation. This highlights the importance of personalized disease modeling in mitochondrial disorders and suggests that effective therapeutic strategies may need to be tailored to individual cellular responses.

**Supplementary Information:**

The online version contains supplementary material available at 10.1038/s41598-026-43696-1.

## Introduction

Mitochondria are best known for their role in cellular energy production through oxidative phosphorylation (OXPHOS). However, their functions extend far beyond ATP synthesis. Mitochondria are central regulators of several critical cellular processes, including the generation of reactive oxygen species (ROS), the control of intrinsic apoptosis, the regulation of autophagy, and the coordination of iron metabolism^1^. Consequently, impairment in mitochondrial function can disrupt a wide range of cellular activities, contributing to the pathogenesis of numerous diseases.

Programmed cell death, or apoptosis, is a tightly regulated process by which cells can eliminate themselves in response to damage or stress. It occurs primarily via two major pathways: the intrinsic (mitochondrial) and extrinsic (death receptor-mediated) pathways^2^. Mitochondria are essential mediators of the intrinsic pathway, where the release of cytochrome c (cyt c) from the mitochondrial intermembrane space into the cytosol leads to the activation of initiator and effector caspases. The B-cell lymphoma-2 (BCL2) family of proteins plays a key regulatory role in this pathway, with pro-apoptotic members (e.g., BCL2 associated X (BAX), BCL2 Antagonist (BAK)) promoting cyt c release, while anti-apoptotic members (e.g., BCL2, B-cell lymphoma-extra large (BCL-xL), Myeloid cell leukemia 1 (MCL1)) inhibitit, thereby modulating cell survival.

The m.3243A>G mutation is one of the most common pathogenic variants in human mitochondrial DNA (mtDNA). Located in the mitochondrial tRNA-Leu(UUR) gene, this heteroplasmic mutation impairs mitochondrial protein synthesis, which can lead to defective OXPHOS, and mitochondrial dysfunction^3^. The clinical manifestations associated with m.3243A>G are remarkably heterogeneous. Patients may present with a neurological disease, mitochondrial encephalomyopathy, lactic acidosis, and stroke-like episodes (MELAS), maternally inherited diabetes and deafness (MIDD), or a range of other cardiac, gastro-intestinal and/or neuromuscular symptoms. While heteroplasmy, i.e. the proportion of mutated mtDNA in patient tissues, contributes to this variability, it does not fully account for the observed phenotypic diversity. Nuclear genetic background, epigenetic influences, and environmental factors are also thought to modulate disease severity and progression^4^.

Given this variability, there is a critical need for patient-specific models that can recapitulate the unique cellular phenotypes associated with m.3243A>G. iPSCs derived from patients’ somatic cells provide a powerful tool to model mitochondrial disease in a controlled, patient-specific context^5^. iPSCs retain the mitochondrial and nuclear genotypes of the donor while allowing investigation into disease mechanisms in various cell types.

In this study, we used iPSCs derived from two patients harboring the m.3243A>G mutation to examine the effects of this mutation on cell growth, mitochondrial function, oxidative stress, and regulated cell death pathways, including apoptosis, autophagy and ferroptosis. By comparing responses across two genetically distinct patients, we aimed to elucidate both common and patient-specific consequences of the m.3243A>G mutation in human iPSCs.

## Materials and methods

### Patients and iPSC culture

The iPSCs used in this study were previously generated from fibroblasts of two patients (P1 and P2) carrying the m.3243A>G mitochondrial DNA mutation^6^. Appropriate ethical approvals were obtained from the Coordinating Ethics Committee of the Helsinki and Uusimaa Hospital District, and all materials were used with informed consent from the patients. All the patient and control samples were kept anonymous throughout the study, and all methods were performed in accordance with the relevant guidelines and regulations.

At the time of sample collection, P1 was a 39-year-old male diagnosed with MIDD and ataxia, with a normal electrocardiography. P2 was a 55-year-old female presenting with MIDD-like symptoms and severe cardiomyopathy. From these patients, iPSC lines with high (> 60–80%) and low (< 10%) heteroplasmy levels were selected for experiments. In addition, mitochondrially targeted transcription activator-like effector nuclease (mitoTALEN) modified cell lines were used to expand the heteroplasmy range. MitoTALENs were originally generated by Yong Fan’s group at The Third Affiliated Hospital of Guangzhou Medical University, Guangzhou, China, and kindly provided for our study^7^. To isolate the effect of the mutation level, separate isogenic control lines were used for each patient. In total, 2 control and 2 mutated iPSC lines were used from P1, and 3 control lines and 3 mutated lines from P2. Mutation loads were analyzed frequently throughout the study. Details of the cell lines and mutation levels are described in Supplementary Fig. 1.

iPSCs were maintained in Essential 8 medium (Thermo Fisher Scientific, Waltham, MA, USA) on growth factor-reduced Matrigel (Corning, New York, NY, USA). All cultures were supplemented with 0.05 mg/mL uridine throughout in vitro maintenance. Cells were passaged every 3–5 days using ethylenediaminetetraacetic acid (EDTA) (Thermo Fisher Scientific, Waltham, MA, USA), and experiments were conducted using cells between passages 20 and 70.

### m.3243A>G mutation load analysis

The heteroplasmy levels of the m.3243A>G mutation were quantified from total DNA using a TaqMan-based quantitative PCR (q-PCR) assay, which measured the relative abundance of the m.3243A and m.3243G alleles (Supplementary Table 1). Reactions were performed with the Maxima Probe/ROX qPCR Master Mix (Thermo Scientific, Waltham, MA, USA) on a StepOnePlus Real-Time PCR system (Applied Biosystems, Waltham, MA, USA).

### Growth assay

iPSCs were seeded with Rho-associated protein kinase (ROCK) inhibitor on Matrigel-coated 24 well plates. After 24 h a “0 h sample” was collected. From there, cells were collected every 24 h for 4 days. Total proteins were isolated with radioimmunoprecipitation assay (RIPA) lysis buffer (Thermo Fisher Scientific, Waltham, MA, USA) and protein concentrations were determined with Bicinchoninic Acid (BCA) protein quantification (Thermo Fisher Scientific, Waltham, MA, USA) according to manufacturer’s protocol. The assay was performed in 3 biological replicates for each cell line.

### Flow cytometry

iPSCs were seeded with ROCK inhibitor on Matrigel-coated 24 well plate 48 h before analysis. Rock inhibitor was withdrawn from the media next day. For analysis, cells were stained as described in Supplementary Table 2. After staining, cells were washed twice with Dulbecco’s Phosphate-Buffered Saline (DPBS), collected with 0,5 mM EDTA and suspended in DPBS (+ Ca, +Mg). Cells were analyzed by Cytoflex S Analyzer (Beckman Coulter, Brea, CA, USA), and the data analyzed using CytExpert (Version 2.3, Beckman Coulter, Brea, CA, USA). Three biological replicates were analyzed for each sample.

### Mitochondrial respiration and glycolysis analysis

Cellular respiration and glycolysis were assessed using the Seahorse XFe96 Bioanalyzer (Agilent Technologies, Santa Clara, CA, USA). iPSCs were seeded on XF96 cell culture microplates (Agilent Technologies, Santa Clara, CA, USA) at a density of 10,000 cells per well in the presence of ROCK inhibitor. ROCK inhibitor was withdrawn the next day. On the day of the assay, the culture medium was replaced with Seahorse XF Dulbecco’s Modified Eagle Medium (DMEM) (Agilent Technologies) supplemented with 1 mM pyruvate, 2 mM glutamine, and 10 mM glucose. Cells were then incubated for 60 min at 37 °C without CO₂ to allow equilibration. Measurements were taken under basal conditions, followed by sequential injections of mitochondrial inhibitors: 1 µM rotenone to inhibit complex I (CI), and 1 µM antimycin A to inhibit complex III (CIII), effectively halting mitochondrial respiration.

After analysis, cells were washed with DPBS, and lysed using RIPA buffer (Thermo Fisher Scientific, Waltham, MA, USA). Total protein concentration was determined using the BCA Protein Assay Kit (Thermo Fisher Scientific, Waltham, MA, USA). All Seahorse data were normalized to total protein content and analyzed using Wave software (version 2.6.1.53, Agilent Technologies). Each measurement was performed in 3 biological replicates.

### Cytochrome c release assay

The Cyt c Release Apoptosis Assay Kit (ab65311, Abcam, Cambridge, UK) was employed to isolate mitochondrial and cytosolic fractions. Cells were first resuspended in a cytosol extraction buffer and incubated on ice for 10 min to allow membrane permeabilization. The cell suspension was then centrifuged at 800 × g for 10 min to remove intact cells and nuclei. The supernatant was then subjected to a second centrifugation at 10,000 × g for 30 min to pellet the mitochondria. The remaining supernatant was collected as the cytosolic fraction, and the pellet as the mitochondrial fraction for Western Blot analysis. The assay was performed in 3 biological replicates. A representative western blot demonstrating successful subcellular fractionation, as indicated by a mitochondrial marker TOM20 and a cytosolic marker β-actin is shown in Supplementary Fig. 2.

### Cell Viability

iPSCs were seeded with ROCK inhibitor on Matrigel-coated 24 well plates. After 24 h ROCK inhibitor was withdrawn. Treatment was started 2 days after plating and cells were collected after 24 h. Total proteins were isolated with RIPA lysis buffer (Thermo Fisher Scientific, Waltham, MA, USA) and protein concentrations determined with BCA protein quantification (Thermo Fisher Scientific, Waltham, MA, USA) according to manufacturer’s protocol. The assay was performed in 3 biological replicates.

### MDA assay

The malondialdehyde (MDA) assay was performed according to the manufacturer’s instructions (ab118970, Abcam, Cambridge, UK). Briefly, iPSCs were seeded on Matrigel-coated 12-well plates in culture medium supplemented with ROCK inhibitor. After 24 h, the medium was replaced with ROCK inhibitor-free medium, and cells were cultured for an additional 24 h.

Cells were homogenized in MDA lysis buffer supplemented with 1% (v/v) butylated hydroxytoluene (BHT) to prevent artificial oxidation. The lysates were sonicated and centrifuged at 13,000 × g for 10 min at 4 °C. A total of 200 µL of the supernatant was mixed with 600 µL of Developer VII reagent, and the samples were incubated at 95 °C for 1 h. After cooling and centrifugation, the clarified supernatant was transferred to a 96-well plate, and absorbance was measured using a ClarioStar microplate reader (BMG Labtech). The assay was performed in 3 biological replicates.

### Western blotting

Total proteins were extracted using RIPA lysis buffer (Thermo Fisher Scientific, Waltham, MA, USA) supplemented with protease and phosphatase inhibitors (Thermo Fisher Scientific, Waltham, MA, USA), following a 15-minute incubation on ice. Protein lysates (10–15 µg per sample) were separated on 10–15% Tris SDS-PAGE gels and transferred onto polyvinylidene difluoride (PVDF) membranes (Thermo Fisher Scientific, Waltham, MA, USA). Membranes were blocked with 5% milk in DPBS containing 0.1% Tween-20 (PBS-T) for 1 h at room temperature, then briefly rinsed and incubated overnight at 4 °C with primary antibodies (Supplementary Table 3) in 5% Bovine Serum Albumin (BSA) in DPBS. The next day, membranes were washed three times with PBS-T and incubated with the appropriate HRP-conjugated secondary antibodies (Supplementary Table 3) for 1–2 h at RT. After three washes with PBS-T, proteins were detected with enhanced chemiluminescence (ECL) substrate (Thermo Fisher Scientific, Waltham, MA, USA) and imaged with the ChemiDoc MP Imaging System (Bio-Rad, Hercules, CA, USA). Intensity was quantified using Image Lab software (version 5.1, Bio-Rad). Each measurement was performed in 3 technical replicates.

### Gene expression

Gene expression analysis was performed using total RNA extracted with TRItidy G reagent (PanReac AppliChem, Barcelona, Spain). For cDNA synthesis, 1000 ng of RNA was reverse transcribed into cDNA, and diluted 1:10 for use in qRT-PCR. PCR was conducted on a QuantStudio™ Real-Time PCR System (Thermo Fisher Scientific, Waltham, MA, USA) using Maxima SYBR Green Master Mix (Thermo Scientific, Waltham, MA, USA). Relative gene expression levels were calculated using the ΔΔCt method. Primer sequences used in the analysis are listed in Supplementary Table 4. Each measurement was performed in 3 technical replicates.

### Statistical analysis

Data were analyzed using GraphPad Prism 10 software (version 10.3.0, GraphPad Software, San Diego, CA, USA). Results are presented as mean ± standard deviation (SD). Statistical significance was assessed using two-way ANOVA followed by Sidak’s multiple comparisons test when comparing mutated cell lines to control cell lines, and two-way ANOVA followed by Tukey’s multiple comparisons test when evaluating treatment effects. A p-value < 0.05 was considered statistically significant.

### Language editing

The authors used ChatGPT (OpenAI, San Francisco, CA) to assist in improving the language of the manuscript. All scientific content and interpretations were developed by the authors.

## Results

### Patient-Specific Effect of the m.3243A>G mutation on Cell Growth

**Fig. 1:**
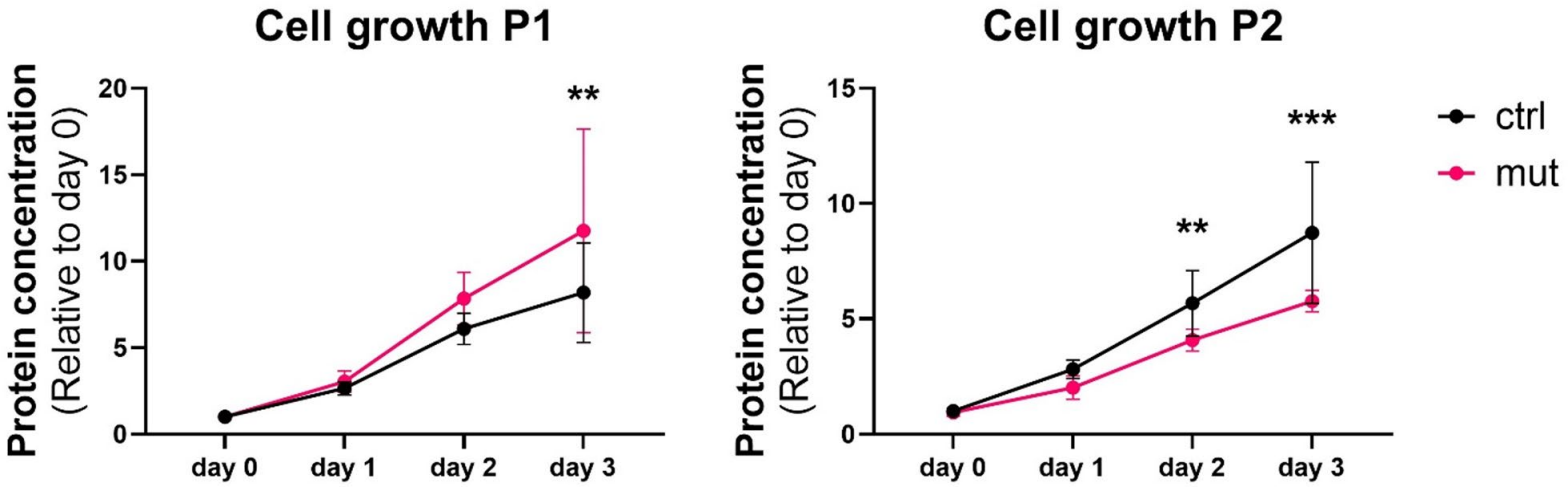
The effect of m.3243 A > G mutations on iPSC growth in P1 and P2. Data are presented as mean ± SD. Statistical analysis was performed using two-way ANOVA followed by Sidak’s multiple comparisons test. **p < 0.01, ***p < 0.001. *n* = 6–9.

IPSCs are highly proliferative and exhibit rapid growth under normal in vitro conditions. However, disruptions in cellular energy metabolism can significantly impair their proliferative capacity, not only due to lack of energy, but also because this may lead to reduced synthesis of key components essential for growth^8^. To assess the impact of the m.3243A>G mutation on iPSC growth, we analyzed changes in total protein levels as a proxy for cell proliferation. Interestingly, P1 iPSCs harboring the m.3243A>G mutation showed a slightly higher growth rate compared to control cells, whereas in P2 cells, cell growth was notably reduced in mutated cells when compared to controls (Fig. 1). The observed differences in cell growth between the two patients led to further studies on interpatient variability in response to the m.3243A>G mutation in iPSCs.

### Patient-Specific Effects of m.3243A>G mutations on Metabolism of iPSCs

**Fig. 2:**
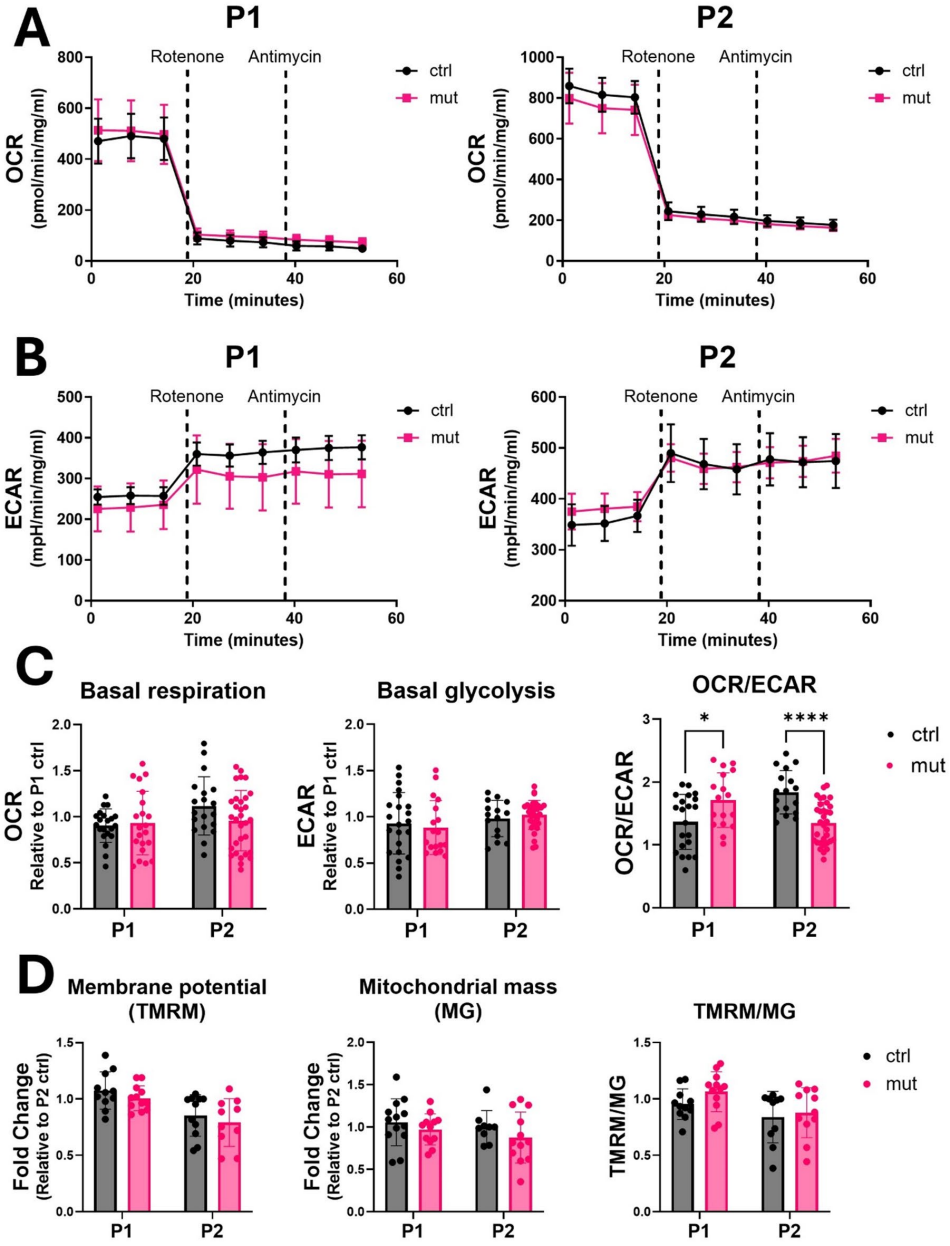
Impact of the m.3243A>G Mutation on Metabolism in Patient-Derived iPSCs. (**A**) Representative oxygen consumption rate (OCR) curves obtained using the Seahorse XF Analyzer for iPSCs derived from Patient 1 (P1) and Patient 2 (P2). (**B**) Representative extracellular acidification rate (ECAR) curves from the Seahorse analysis. (**C**) Quantification of Seahorse data, including basal respiration and glycolysis, in control and m.3243A>G mutated iPSCs. (**D**) Flow cytometry analysis of mitochondrial membrane potential using tetramethylrhodamine methyl ester (TMRM) and mitochondrial mass using Mitotracker Green (MG). The TMRM/MG ratio is shown as an indicator of membrane potential relative to mitochondrial content. Data are presented as mean ± SD, *n* = 10–15. Statistical analysis was performed using two-way ANOVA followed by Sidak’s multiple comparisons test. *p < 0.05, ****p < 0.0001.

Since the m.3243A>G is a mitochondrial DNA mutation, we first assessed its impact on cell respiration in the patient-derived iPSCs. Mitochondrial activity was evaluated using the Seahorse assay, a widely used method for measuring OXPHOS and glycolysis. The analysis revealed no significant m.3243A>G dependent changes in basal respiration or glycolytic activity in either patient’s iPSCs (Fig. 2A, B,C). Furthermore, measurements following rotenone and antimycin A exposure revealed no significant differences between CI or CII dependent respiration among the cell lines (Supplementary Fig. 3A). However, when comparing the balance between respiration and glycolysis, a significant patient-specific shift towards glycolytic activity was seen in iPSCs from P2, whereas in P1, the metabolism surprisingly shifted slightly towards oxidation (Fig. 2C).

To investigate mitochondrial integrity, we measured mitochondrial membrane potential, a key indicator of mitochondrial health. The results showed no significant changes in membrane potential in mutated iPSCs from either patient, even when normalized to mitochondrial mass (Fig. 2D). Mitochondrial mass, assessed by MitoTracker Green staining, was also unchanged, suggesting that m.3243A>G mutations do not alter mitochondrial abundance in iPSCs. To examine whether the observed metabolic changes were associated with altered glucose utilization, we analyzed glucose uptake using the fluorescent glucose analog 2-NBDG and flow cytometry. No significant differences in glucose uptake were observed between mutated and control lines (Supplementary Fig. 3B).

Taken together, these findings show that the m.3243 A > G mutation does not cause substantial mitochondrial dysfunction in patient-derived iPSCs, however subtle patient-specific metabolic responses are evident.

### Dissecting Apoptotic Pathways in m.3243A>G Mutated iPSCs Reveals Unexpected Regulatory Patterns

**Fig. 3:**
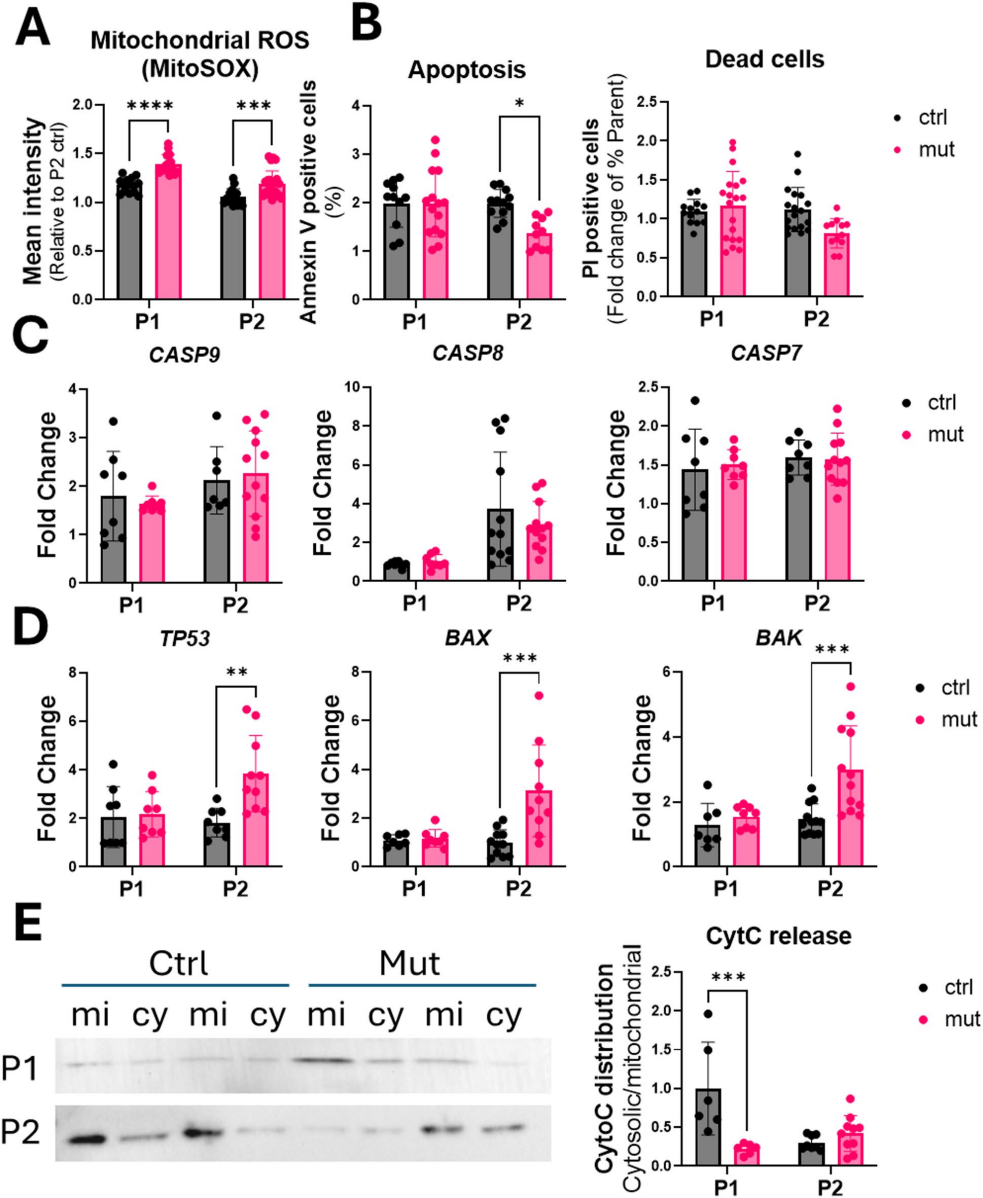
Analysis of Apoptotic Pathways in m.3243A>G mutated iPSCs. (**A**) Flow cytometry analysis of mitochondrial ROS production using MitoSOX staining. Representative flow cytometry dot plots and histograms in Supplementary Fig. 4. (B) Quantification of apoptotic and dead cells by Annexin V/propidium iodide (PI) double staining and flow cytometry. Representative flow cytometry dot plots and histograms in Supplementary Fig. 5. (C) Relative gene expression levels of caspases involved in apoptosis initiation and execution (*CASP8*, *CASP9*, and *CASP7*), as measured by qPCR. (D) Gene expression of proapoptotic regulators *TP53*, *BAX*, and *BAK*. (E) Western blot analysis of cyt c distribution between the cytosolic and mitochondrial fractions, indicating mitochondrial outer membrane permeabilization status. Original blots are presented in Supplementary Fig. 11. Data are presented as mean ± SD, *n* = 6–9. Statistical analysis was performed using two-way ANOVA followed by Sidak’s multiple comparisons test. *p < 0.05, **p < 0.01, ***p < 0.001, ****p < 0,0001.

Since the m.3243A>G mutation did not result in significant OXPHOS dysfunction, we explored alternative mechanisms that might contribute to altered cell growth. Mitochondria are key regulators of ROS production, and respiratory chain dysfunction is known to elevate mitochondrial ROS levels^9^. Flow cytometry analysis revealed that mitochondrial ROS levels were increased in iPSCs from both patients carrying the m.3243A>G mutations (Fig. 3A, Supplementary Fig. 4). Elevated ROS can induce apoptosis, which could potentially explain the reduced cell proliferation^9^. To assess this, we analyzed apoptosis using Annexin V/PI staining and flow cytometry. Surprisingly, apoptosis was significantly decreased in mutated iPSCs from P2 (Fig. 3B, Supplementary Fig. 5).

To better understand the mechanisms underlying reduced apoptosis, we analyzed the expression of caspase (CASP) genes using qRT-PCR. *CASP9* and *CASP8* serve as initiators of the intrinsic and extrinsic apoptosis pathways, respectively, while *CASP7* functions as an executioner caspase. The m.3243A>G mutations did not significantly alter the expression of any of these caspase genes. However, *CASP8* expression was significantly higher in all iPSCs from P2 when compared to P1 cells (Fig. 3C).

Given the central role of mitochondria in the intrinsic apoptotic pathway, we further examined the expression of key pro-apoptotic genes. The transcription factor p53 promotes apoptosis through regulation of genes for BAX and BAK, which are localized to the mitochondrial outer membrane and facilitate cyt c release. Surprisingly, expression levels of *P53*, *BAX*, and *BAK* were significantly upregulated in P2 mutated iPSCs (Fig. 3D). Since these proteins are involved in mitochondrial outer membrane permeabilization and subsequent cyt c release, we further quantified cyt c levels in the cytosol and in mitochondria. Interestingly, despite high expression of these genes, the m.3243A>G mutations did not induce cyt c release in P2 iPSCs. In contrast, mutated iPSCs from P1 exhibited reduced cytosolic to mitochondrial cyt c ratio when compared to control cells (Fig. 3E, Supplementary Fig. 2).

We further measured the expression of key components of the extrinsic apoptosis pathway, including Death receptor 4 (DR4) and Fas Cell Surface Death Receptor (FAS). While *DR4* expression remained unchanged across all lines, *FAS* expression was notably reduced in P2 mutated iPSCs, suggesting a potential downregulation of extrinsic apoptotic signaling in these lines (Supplementary Fig. 6).

Together these results show that despite increased expression of pro-apoptotic genes, apoptosis is downregulated in P2 iPSCs with m.3243 A > G mutations.

### Upregulation of Anti-Apoptotic BCL2 Family Proteins Confers Apoptosis Resistance in P2 m.3243A>G iPSCs

**Fig. 4:**
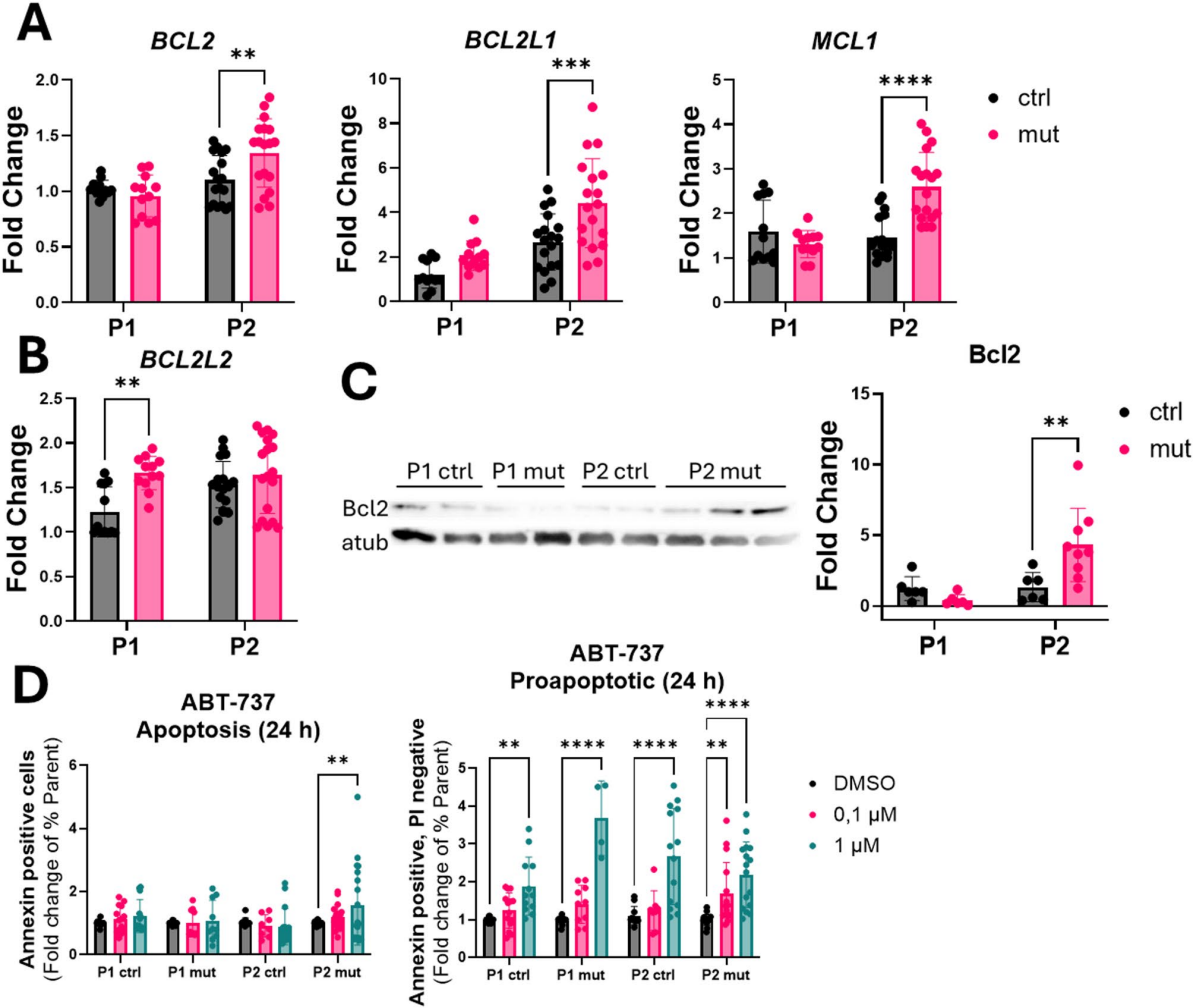
Regulation of Apoptosis by BCL2 Family Proteins in m.3243A>G iPSCs. (A, B) Relative gene expression levels of anti-apoptotic BCL2 family members (*BCL2*,* BCL2L1*,* MCL1*, and *BCL2L2*) in control and m.3243A>G mutated iPSCs, measured by qPCR. (C) Western blot analysis of BCL2 protein expression in control and mutated iPSCs. Original blots are presented in Supplementary Fig. 11. (D) Quantification of apoptosis by Annexin V/PI staining and flow cytometry after 24-hour treatment with ABT-737 at 0.1 or 1 µM. Data are presented as mean ± SD, *n* = 6–9. Statistical analysis was performed using two-way ANOVA followed by Sidak’s multiple comparisons test (A, B & C) or Tukey’s multiple comparisons test (D). *p < 0.05, **p < 0.01, ***p < 0.001, ****p < 0,0001.

BAX and BAK are pro-apoptotic proteins located on the mitochondrial outer membrane that promote apoptosis upon activation^10^. Their activity is tightly regulated by anti-apoptotic members of the BCL2 protein family, which inhibits them through direct binding. Given that *BAX* and *BAK* expressions were significantly upregulated in P2 mutated iPSCs, yet apoptosis was decreased, we next examined whether the anti-apoptotic BCL2 family members might be involved in this phenomenon. Gene expression analysis revealed that *BCL2*,* BCL2L1* (encoding BCL-xL), and *MCL1* were significantly upregulated in P2 mutated iPSCs (Fig. 4A). In contrast, *BCL2L2* was elevated only in P1 mutated cells (Fig. 4B). Consistent with gene expression data, BCL2 protein levels were also significantly increased in P2 mutated iPSCs (Fig. 4C). These findings suggest that upregulation of anti-apoptotic BCL2 family proteins may underlie the suppression of apoptosis in P2 mutated iPSCs, despite the elevated expression of pro-apoptotic genes.

Further supporting this, gene expression of two additional apoptosis-regulating factors, BCL2 Associated Agonist Of Cell Death (BAD) and Forkhead box O1 (FOXO1), was decreased in P2 mutated cells, while both genes were upregulated in P1 mutated lines when compared to controls (Supplementary Fig. 7). BAD is a pro-apoptotic member of the BCL2 family that antagonizes BCL2 and BCL-xL, while FOXO1 is a transcription factor that promotes the expression of apoptosis-related genes. Downregulation of these genes in P2 further supports the suppression of apoptotic signaling, despite the upstream activation.

To functionally verify the role of anti-apoptotic signaling in apoptosis suppression in P2, we evaluated the effects of pharmacological inhibition of BCL2 family members on apoptosis. For this we selected a small-molecule ABT-737, a selective inhibitor of BCL2 and BCL-xL.

We first tested ABT-737 at 0.1 µM, 1 µM, and 5 µM concentrations over a 24-hour period. Cytotoxicity assays indicated that 5 µM concentration was significantly toxic to all cell lines (Supplementary Fig. 8) and was therefore excluded from subsequent assays. While 1 µM ABT-737 also reduced cell viability across all iPSC lines, the 0,1 µM concentration reduced viability only in P1 mutated cells (Supplementary Fig. 8). In apoptosis assays, the 1 µM ABT-737 elevated proportion of pro-apoptotic cells in all the lines but significantly increased apoptotic cell death only in P2 mutated iPSCs (Fig. 4D). Lower concentration, 0.1 µM ABT-737, increased the pro-apoptotic cell population exclusively in P2 mutated iPSCs.

Taking together, these results show that P2 mutated iPSCs are more sensitive to apoptosis induction by BCL2 family inhibition than the other cell lines, suggesting that the BCL2 family is involved in suppressing apoptosis in this patient.

### The m.3243A>G induces No Evident Changes in Autophagy

Autophagy is a critical cellular mechanism for the removal of excess or damaged organelles and plays a protective role under stress conditions, including by modulating apoptosis^11^. Since enhanced autophagy can suppress apoptotic responses, we investigated whether autophagy was altered in our m.3243A>G mutated iPSCs.

**Fig. 5:**
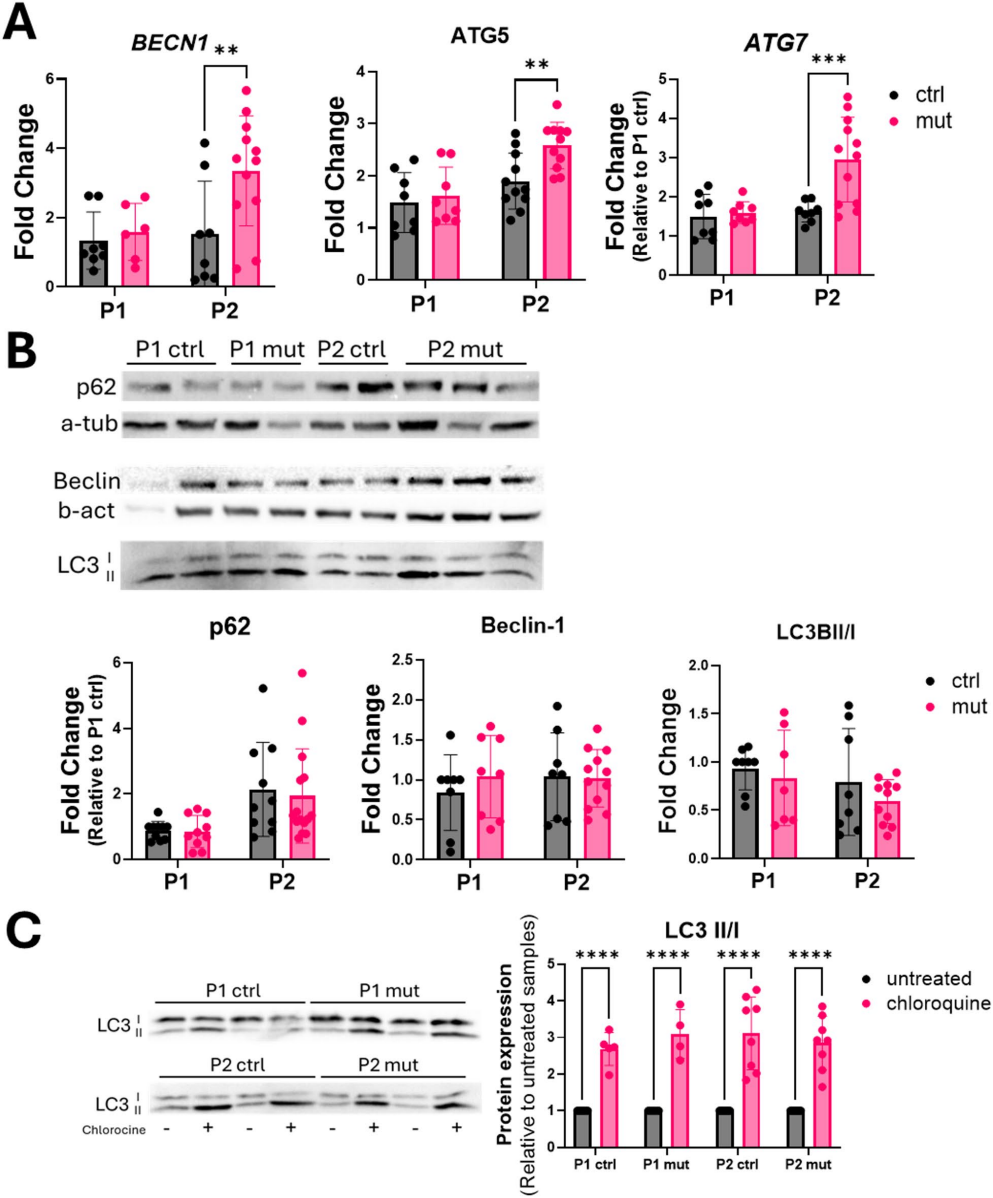
Autophagy-Related Markers in m.3243A>G iPSCs. (**A**) Relative gene expression of key autophagy regulators *BECN1* (Beclin-1), *ATG5*, and *ATG7* in control and m.3243A>G mutated iPSCs, measured by qPCR. (**B**) Western blot analysis of autophagy-related proteins p62, Beclin-1, and LC3B (LC3B-I and LC3B-II). a-tubulin (atub) and b-actin (b-act) were used as loading controls. Original blots are presented in Supplementary Fig. 12. (C) Western blot analysis of LC3B-II/LC3B-I ratio in iPSCs with and without chloroquine, to assess autophagic flux. Original blots are presented in Supplementary Fig. 13. Data are presented as mean ± SD, *n* = 6–9. Statistical analysis was performed using two-way ANOVA followed by Sidak’s multiple comparisons test. **p < 0.01, ***p < 0.001, ****p < 0,0001.

Gene expression analysis revealed that the expression of key autophagy-related genes, Beclin-1 (*BECN1*) and Autophagy protein 5 and 7 (*ATG5*, ATG7), was significantly upregulated in P2 mutated iPSCs (Fig. 5A). To determine whether these transcriptional changes were reflected at the protein level, we examined the expression of major autophagy markers including p62, Beclin-1, and LC3B-II by Western blot. Surprisingly, we observed no significant differences in the protein levels of these markers between mutated and control iPSCs (Fig. 5B). To resolve the discrepancy between gene and protein expression, we assessed autophagic flux by pharmacologically inhibiting lysosomal degradation using chloroquine and measuring the resulting accumulation of LC3B-II. Following chloroquine treatment, we observed a clear and comparable increase in LC3B-II levels in all iPSC lines when compared to untreated controls, indicating active and equal autophagic flux in all lines (Fig. 5C).

These results suggest that although autophagy-related gene expression is elevated in P2 mutated iPSCs, the m.3243A>G mutation does not affect autophagic flux at functional level.

### P2 Mutated iPSCs Exhibit Signs of Increased Ferroptotic Susceptibility

Apoptosis is not the only form of regulated cell death. Ferroptosis is a distinct form of cell death driven by iron accumulation, which promotes the generation of ROS and subsequent lipid peroxidation^12^. Given the significant reduction in apoptosis observed in our m.3243A>G mutated iPSCs, along with slow cell proliferation, we explored whether ferroptosis might be altered in these cells.

**Fig. 6:**
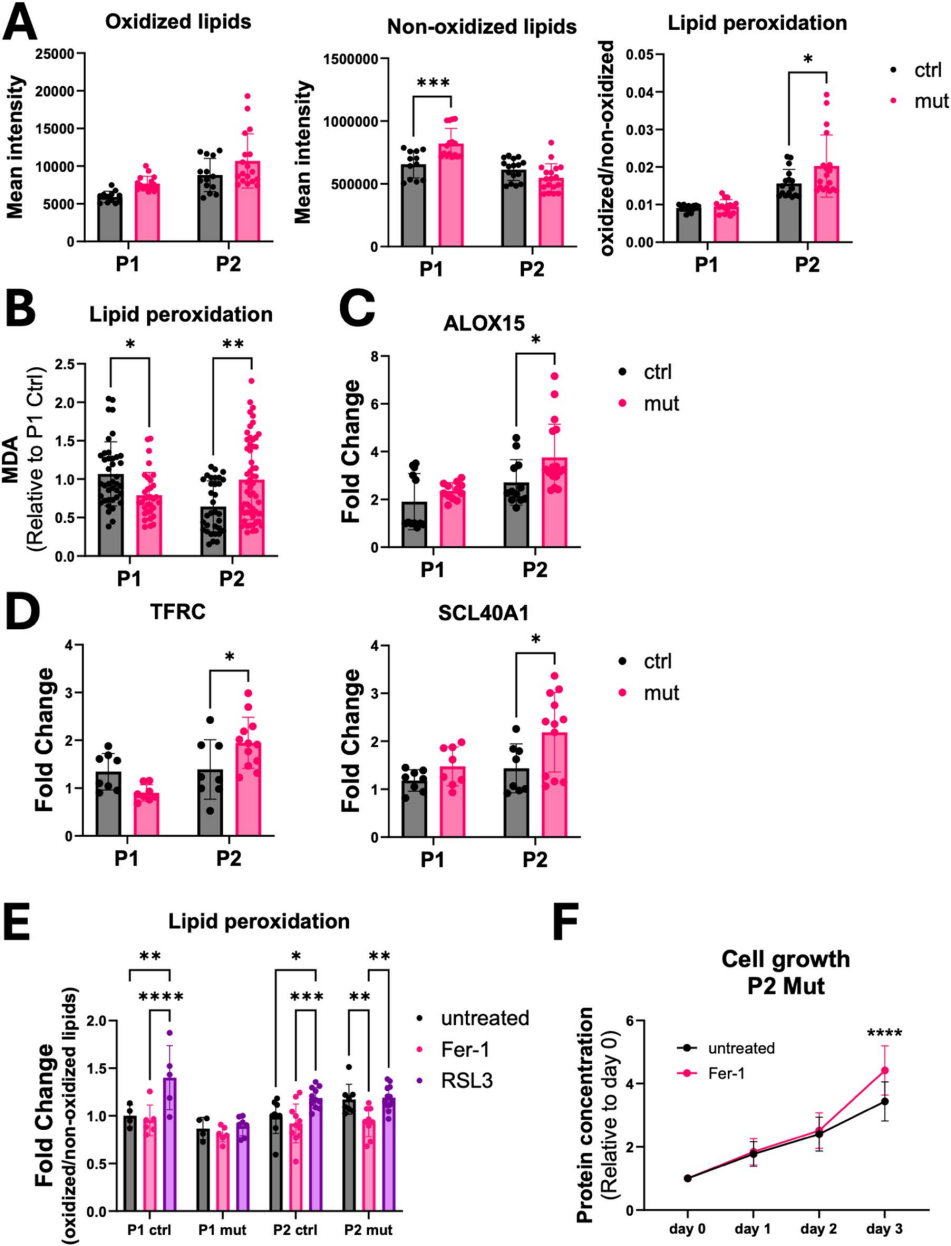
Effects of the m.3243A>G Mutation on Ferroptosis-Associated Markers in iPSCs. (**A**) Flow cytometry analysis using BODIPY 581/591 C11 staining to quantify oxidized and non-oxidized lipids, and their ratio, in control and m.3243A>G mutated iPSCs. (**B**) Malondialdehyde (MDA) assay to assess lipid peroxidation levels as an indicator of ferroptosis. (**C**) Gene expression analysis of lipid peroxidation-related enzyme *ALOX15*. (D) Gene expression of iron metabolism regulators *TFRC* (transferrin receptor) and *SLC40A1 (*ferroportin), both involved in iron uptake and export, respectively. (E) BODIPY 581/591 C11 staining analyzed by flow cytometry was used to quantify oxidized lipids relative to non-oxidized lipids 24 h after treatment with 2 µM ferrostatin-1 (Fer-1) or 100 nM RSL3. (F) Effect of 2 µM Fer-1 treatment on mutated P2 cell growth over a 3-day period. Data are presented as mean ± SD, *n* = 6–9. Statistical analysis was performed using two-way ANOVA followed by Sidak’s multiple comparisons test. *p < 0.05, **p < 0.01, ***p < 0.001, ****p < 0,0001.

To assess lipid peroxidation, we performed BODIPY 581/591 C11 flow cytometry analysis to quantify both total and oxidized lipid species (Fig. 6A). In P1 mutated iPSCs, we observed an increase in total lipid content when compared to controls. In contrast, P2 mutated iPSCs exhibited an elevated ratio of oxidized to non-oxidized lipids, suggesting enhanced lipid peroxidation relative to controls.

To evaluate more advanced stages of lipid oxidation, we measured levels of MDA, which is a byproduct of polyunsaturated fatty acid (PUFA) peroxidation. MDA levels in P2 mutated iPSCs were increased, while P1 mutated iPSCs displayed a reduction in MDA levels (Fig. 6B). To further explore this pathway, we examined the expression of arachidonate 15-lipoxygenase (ALOX15), an enzyme that catalyzes lipid peroxidation and promotes ferroptosis. *ALOX15* expression was markedly upregulated in P2 mutated iPSCs (Fig. 6C), reinforcing the evidence of heightened ferroptotic activity in these cells.

Because iron metabolism is central to ferroptosis, we also analyzed the expression of key iron transport genes. Both transferrin receptor (*TFRC*) and ferroportin (*SLC40A1*) were upregulated in P2 mutated iPSCs, consistent with an enhanced ferroptotic phenotype (Fig. 6D).

Given the central role of glutathione in neutralizing lipid peroxides, we analyzed the expression of Glutamate-Cysteine Ligase Catalytic Subunit (*GCLC*) and Glutamate-Cysteine Ligase Modifier Subunit (*GCLM*) genes, key components of glutamate-cysteine ligase (GCL), the rate-limiting enzyme in glutathione biosynthesis^13^. *GCLC* and *GCLM* were upregulated in P2 mutated iPSCs, suggesting a compensatory antioxidant response. However, expression levels of other Nuclear factor erythroid 2-related factor 2 (NRF2)-regulated antioxidant genes, NAD(P)H dehydrogenase [quinone] 1 (*NQO1*) and heme oxygenase 1 gene (*HMOX1*), remained unchanged (Supplementary Fig. 9), implying that the broader NRF2-mediated oxidative stress response was not robustly activated.

To assess the contribution of ferroptosis to decreased cell proliferation, cells were treated with ferrostatin-1 (Fer-1), a lipophilic antioxidant that inhibits ferroptosis by preventing iron-dependent lipid peroxidation. Notably, treatment with 2 µM Fer-1 selectively reduced lipid peroxidation in P2 mutated cells after 24 h (Fig. 6E) and this was associated with increased cell growth after 3 days of treatment (Fig. 6F), an effect also observed exclusively in the mutated P2 lines (Supplementary Fig. 10). We further induced ferroptosis using 100 nM RSL3, a direct inhibitor of glutathione peroxidase 4 (GPX4), a key enzyme that protects cells from lipid peroxidation. Interestingly, RSL3 increased lipid peroxidation significantly only in control cells (Fig. 6E), suggesting inactive GPX4 in the mutated iPSCs from both patients.

Together, these findings suggest that increased ferroptotic susceptibility, characterized by elevated lipid peroxidation and increased expression of iron transport-related genes, is responsible for the decreased cell proliferation in mutated P2 iPSCs.

## Discussion

Mitochondrial diseases exhibit a striking degree of clinical heterogeneity, with symptoms ranging from asymptomatic to life-threatening. This variability is exemplified in patients carrying the m.3243A>G mutation. While heteroplasmy is a key determinant of disease severity, numerous studies have shown that it alone cannot fully account for the diversity of clinical outcomes. Nuclear genetic background, environmental stressors, and epigenetic modulation likely play additional roles in shaping phenotype expression.

In this study, we used patient-derived iPSCs from two individuals harboring the m.3243A>G mutation to investigate mitochondrial function, cell death pathways, and stress responses. iPSCs offer a unique advantage as they allow for patient-specific modeling in various cell types including a pluripotent, non-terminally differentiated and highly proliferative cellular state. IPSCs predominantly rely on glycolysis rather than oxidative phosphorylation for energy production, which limits their ability to recapitulate mitochondrial bioenergetic defects observed in metabolically demanding tissues. Although iPSCs retain functional mitochondria and are capable of mounting mitochondrial stress responses^14^, the extent to which these responses reflect disease-relevant mechanisms in differentiated cells remains uncertain. Thus, our findings in iPSCs may not be directly translatable to other cell types. Given the strong tissue specificity of mitochondrial diseases, further validation in disease-relevant cell types will be essential to determine the physiological and pathological relevance of these observations.

Our data reveal patient-specific differences in cellular responses, despite similar levels of the m.3243A>G mutation, supporting the idea that factors beyond heteroplasmy contribute significantly to phenotype variability, and consistent with previous reports highlighting the importance of the nuclear genetic background in modulating the effects of this mutation in cellular models^15,16^. In particular, P2 mutated iPSCs showed reduced proliferation and a subtle shift towards glycolytic metabolism. Surprisingly, in contrast, P1 mutated iPSCs displayed a shift towards mitochondrial respiration and slightly enhanced cell growth. The observed metabolic shifts were very subtle but suggest different compensatory responses to mitochondrial dysfunction in the two patients.

One of the most striking findings of our study was the paradoxical regulation of apoptosis in P2 mutated cells. Although we observed upregulation of intrinsic pro-apoptotic genes (*TP53*, *BAX*, *BAK*), the actual rate of apoptosis was significantly reduced. This apparent contradiction was accompanied by increased expression of anti-apoptotic BCL2 family members at both the gene and protein levels. The overexpression of BCL2 in particular is notable, as it has been widely documented in cancer models where it confers resistance to apoptosis by inhibiting cyt c release and preserving mitochondrial membrane integrity^17^. This same mechanism likely underlies the apoptosis resistance seen in P2 mutated cells as the functional importance of BCL2 in P2 iPSCs was verified by their high sensitivity to BCL2 inhibition. Some studies have shown that BCL2 overexpression can prevent mitochondrial dysfunction^18,19^. On the other hand, when BCL2 rescues the membrane potential of mitochondria, it can prevent the breakdown of dysfunctional mitochondria and promote their accumulation in the cell, which could explain why we were not able to detect a difference in mitochondrial membrane potential while cell growth was hampered, and oxidative stress increased.

Given the confounding proliferation and apoptosis data, we next explored autophagy and ferroptosis as alternative mechanisms of cellular quality control and death. Although autophagy-related genes were upregulated in P2 mutated iPSCs, autophagic flux remained unchanged, suggesting that the mRNA changes did not translate into functional activation of the pathway. Transcript level changes do not always result in functional effects and a possible explanation for this here is the concurrent upregulation of BCL2, which is known to inhibit not only apoptosis but also autophagy by binding to key autophagy regulators such as Beclin-1^20^. This dual inhibitory role of BCL2 may therefore contribute to the observed disconnect between autophagy-related gene expression and autophagic activity in P2 mutated cells. However, while we cannot exclude the role of autophagy in m.3243A>G disease, it does not seem to play a significant role in iPSCs.

The expression of genes involved in lipid peroxidation and iron metabolism, key hallmarks of ferroptosis, was also significantly elevated in P2 mutated iPSCs. Importantly, this transcriptional upregulation was accompanied by a functional increase in lipid peroxidation, supporting enhanced ferroptotic activity in these cells. While mitochondrial ROS levels were elevated in both P1 and P2 mutated iPSCs, lipid peroxidation was only increased in P2, indicating patient-specific differences in susceptibility to oxidative damage. This result was further supported by the observation that Fer-1, which reduces lipid peroxidation, significantly decreased lipid peroxidation only in P2 mutated cells. Further, the Fer-1 treatment enhanced cell proliferation in P2 cells with high mutation levels, suggesting that increased ferroptosis underlies the growth defect in these cells.

This discrepancy in lipid peroxidation between the two patients suggests that additional regulatory factors may modulate the cellular response to ROS. One possibility is the antioxidant defense system, particularly glutathione (GSH), which plays a central role in detoxifying lipid peroxides via the GPX4 pathway[13]. GCLC and GCLM, encoding the catalytic subunits of GCL, the rate-limiting enzyme in GSH biosynthesis, were upregulated in P2 mutated iPSCs, likely as a compensatory response to rising oxidative stress. However, the failure to contain lipid peroxidation suggests this defense is insufficient.

Interestingly, inhibition of GPX4 increased lipid peroxidation in control cells but had little effect in mutated cells from either patient. This observation may indicate that GPX4 is already partially compromised in the mutated cells, and thus further inhibition does not produce a detectable response. Such deficit in GPX4 activity could also explain why the observed upregulation of GCLC and GCLM does not appear sufficient to mitigate lipid peroxidation in P2 mutant cells.

This insufficiency could result from either excessive lipid peroxide production or limitations in GSH biosynthesis. The latter is particularly plausible, as this GPX4 deficit was seen in both patients’ cells with high mutation levels. Further, cysteine, a critical precursor for GSH, is generated via the mitochondrial-dependent transsulfuration pathway^21^. Therefore, impaired mitochondrial function may hinder effective GSH synthesis despite increased GCLC and GCLM expression. This deficit could leave the cells vulnerable to lipid peroxidation and ferroptosis. Further mechanistic studies are needed to explore this possibility in detail.

Notably, P2 iPSCs were derived from a patient with severe cardiomyopathy. Previous studies have shown that OXPHOS dysfunction in cardiac tissue predisposes cardiomyocytes to ferroptosis^22^. In this context, our results support the notion that ROS-mediated lipid peroxidation and iron dysregulation may contribute to cardiac pathology in mitochondrial disease, particularly when apoptosis is suppressed through upregulation of anti-apoptotic factors such as BCL2. However, further investigation using more disease-relevant cell types, such as cardiomyocytes, is necessary to substantiate this mechanism.

In addition to the limitations inherent to the relevance of the iPSC model, this study has also other constraints. First, the small sample size, limited to two patients, precludes drawing broad conclusions about the effects of the m.3243A>G mutation across larger patient populations. However, the study design relies on comparisons between mutated and isogenic control cells rather than direct comparisons between patient-derived lines, thereby focusing on the impact of the mutation itself within a controlled genetic background. Secondly, line specific variation is known to exist between individual iPSC clones. To account for this, multiple independent iPSC clones have been analyzed for each group in all experiments.

While not able to explain the clinical heterogeneity seen between different patients with the m.3243A>G mutation, our study clearly shows that the heteroplasmy level alone cannot account for the varying phenotypes, and underlying genetic background has a major role in dictating the disease outcome. Based on the genetic background, the cells respond differently to the same initial insult, i.e. OXPHOS defect and/or oxidative stress, and employ alternative tissue specific metabolic and stress responses that can either ameliorate or aggravate the defect. One of these differentially regulated pathways could be preference for a specific cell death pathway.

## Conclusion

Collectively, our results highlight that the m.3243A>G mutation can exert highly divergent cellular effects, even in the same cell type, depending on patient context. While traditional models have emphasized OXPHOS deficiency and heteroplasmy as primary disease drivers, our findings underscore the importance of additional pathways, including BCL2-mediated apoptosis resistance and ferroptosis, in mitochondrial disease pathogenesis. Patient-derived iPSC models provide a powerful tool for dissecting these individual responses and could aid in developing personalized therapeutic approaches.

## Supplementary Information

Below is the link to the electronic supplementary material.

Supplementary Material 1

## Data Availability

All the data is included in the manuscript and supplementary data.
