# Supplementary material for: Patient specific regulation of metabolism and cell death pathways in m.3243A>G mutated iPS-cells
Source: Sci Rep. 2026 Apr 27;16:19744. doi: 10.1038/s41598-026-43696-1 (PMC13315276; doi:10.1038/s41598-026-43696-1)
Supplement: Supplementary file 1 — Supplementary Material 1 [file 41598_2026_43696_MOESM1_ESM.docx]

# **Patient Specific Regulation of Metabolism and Cell Death Pathways in m.3243A>G Mutant iPS-cells**

**Supplementary Data**

Sanna Ryytty ^1^, Katriina Nurminen ^1^, Teemu Tiukuvaara^1^, Petri Mäkinen ^1^, Anu Suomalainen ^2,3^, Riikka H. Hämäläinen ^1^*

Supplementary Figure 1. Quantitative PCR analysis of m.3243A>G mutation load in the iPSC lines used in this study. Lines with low mutation load (<10%) were used as controls (Ctrl), while lines with high mutation load (>60%) served as mutant lines (Mut). Three technical replicates per line. In patient 2, the Ctrl3 and Mut2 lines were generated from the Mut3 line using mitoTALENs. P1/P2 = Patient 1 or 2

*
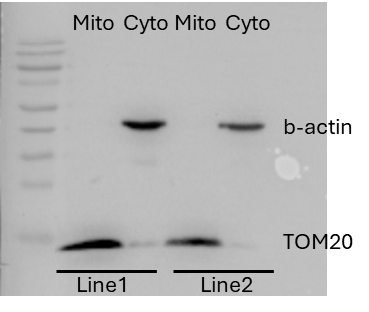
*

Supplementary Figure 2. A representative western blot demonstrating successful subcellular fractionation, as indicated by the mitochondrial marker TOM20 and the cytosolic marker β-actin. Mito = mitochondrial sample, Cyto = Cytosolic sample.


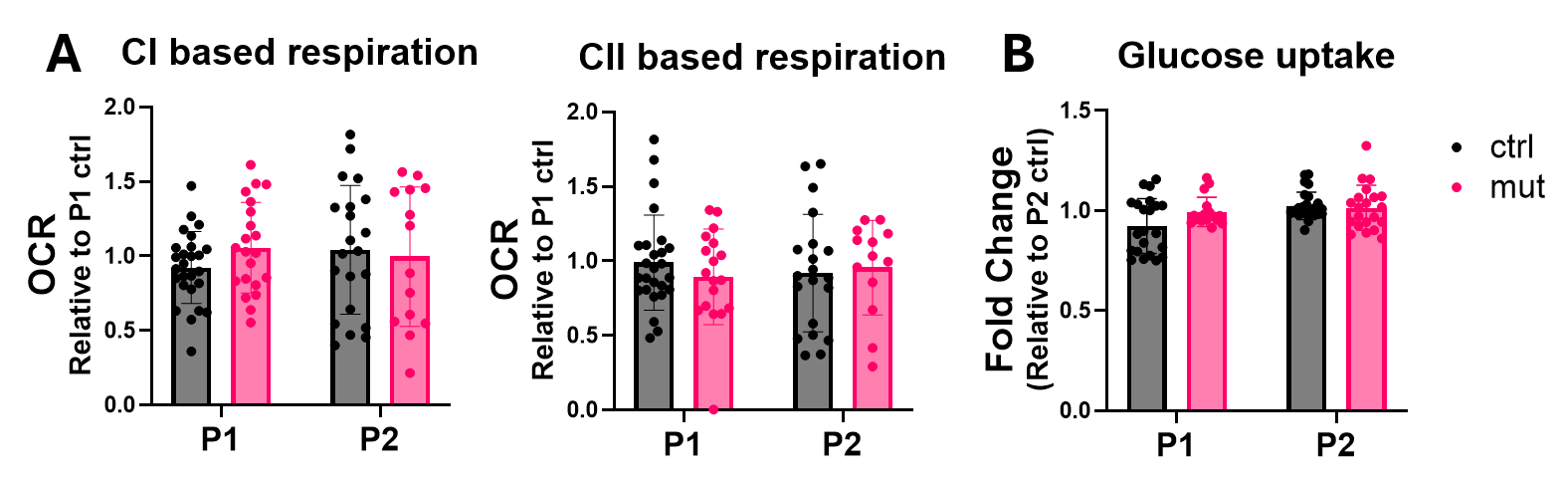


Supplementary Figure 3. (A) Seahorse XFe96 analysis of oxygen consumption rate (OCR), assessing mitochondrial respiration driven by CI and CII. (B) Glucose uptake quantified by flow cytometry using the fluorescent glucose analog 2-NBDG. Data are presented as mean ± SD, n=10-15.


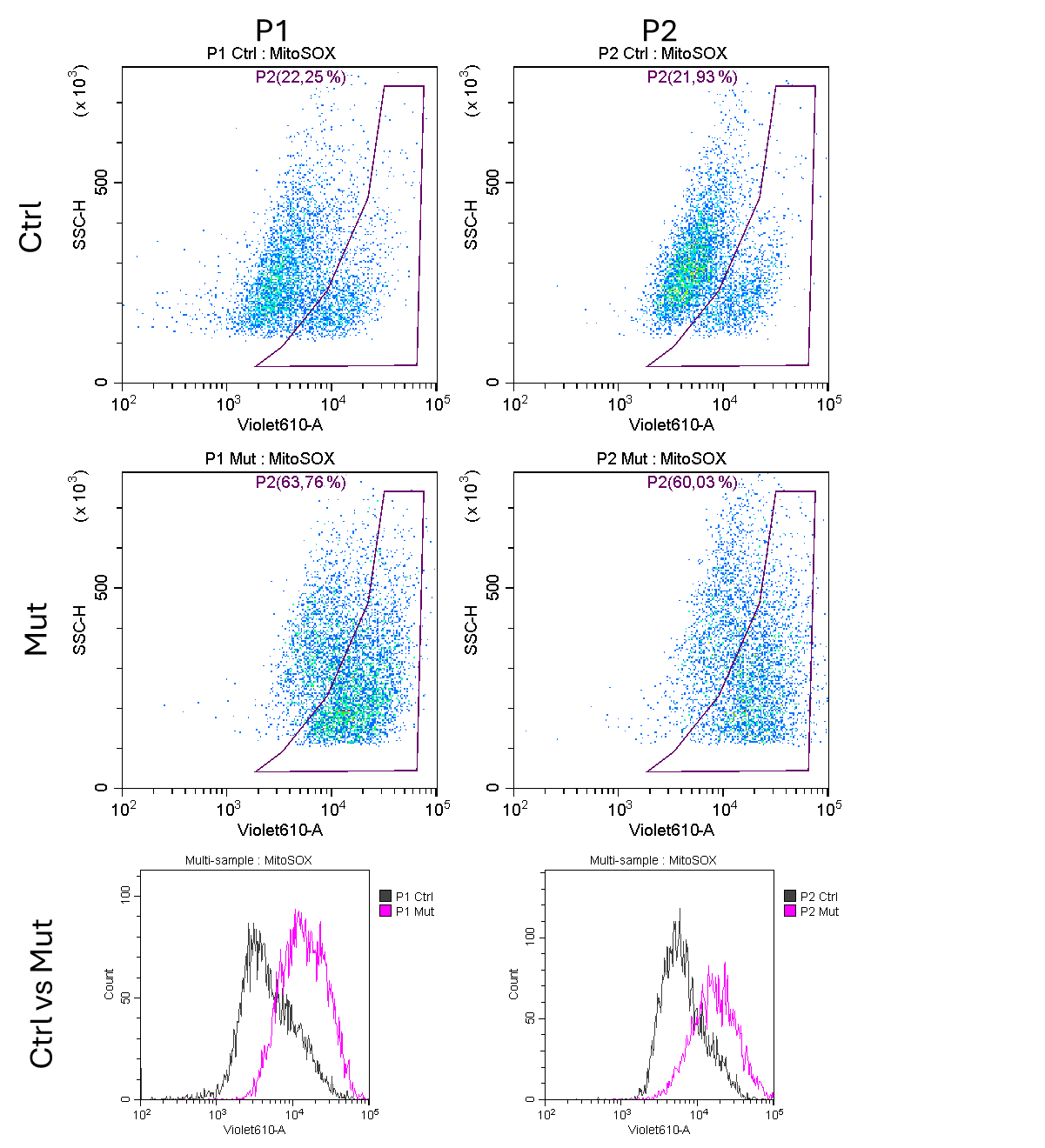


Supplementary Figure 4. Representative flow cytometry dot plots and overlaid histograms for MitoSOX staining. Violet610=MitoSOX.


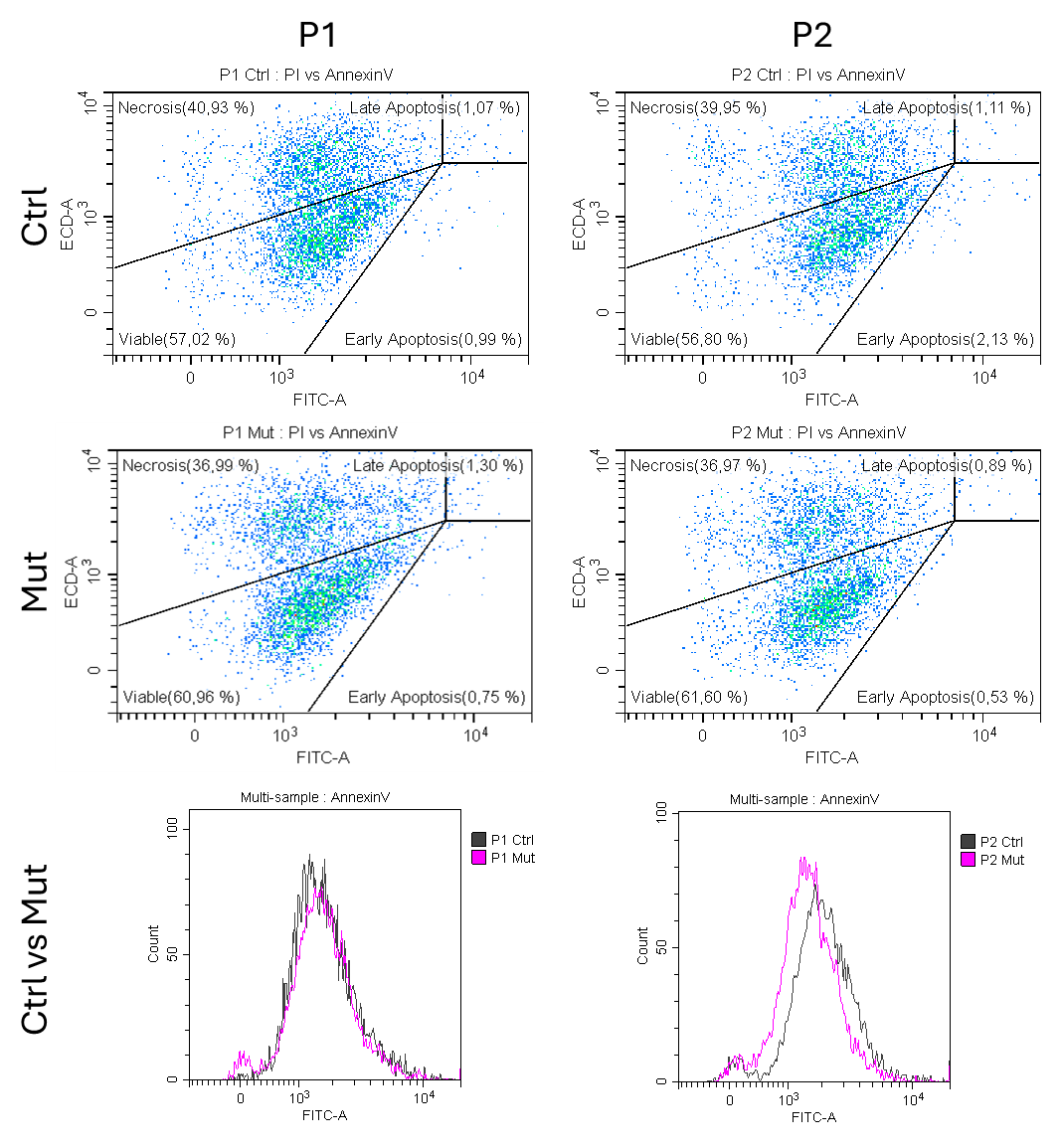


Supplementary Figure 5. Representative flow cytometry dot plots and overlaid histograms for Annexin V and PI staining. ECD-A = PI, FITC-A = Annexin V


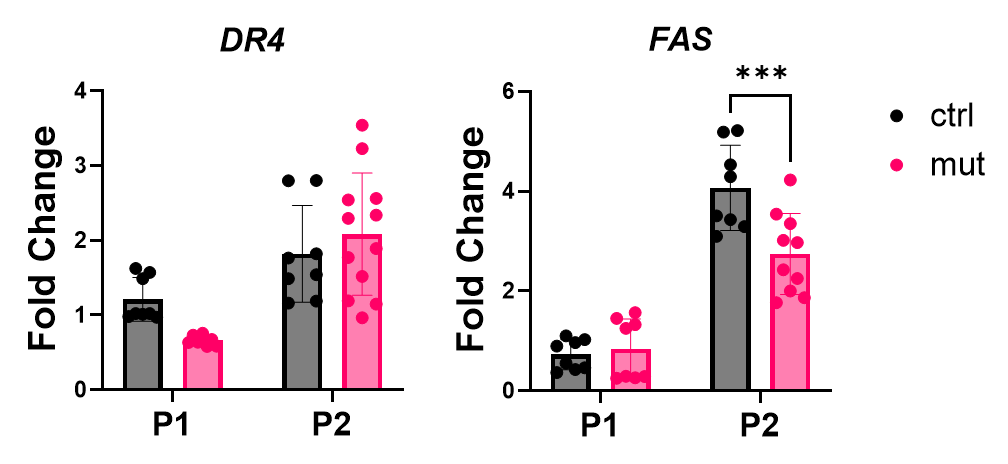


Supplementary Figure 6. Gene expression of the death receptors *DR4* and *FAS* of the extrinsic apoptotic pathway. Data are presented as mean ± SD, n=6-12. Statistical analysis was performed using two-way ANOVA followed by Sidak’s multiple comparisons test. ***p < 0.001.


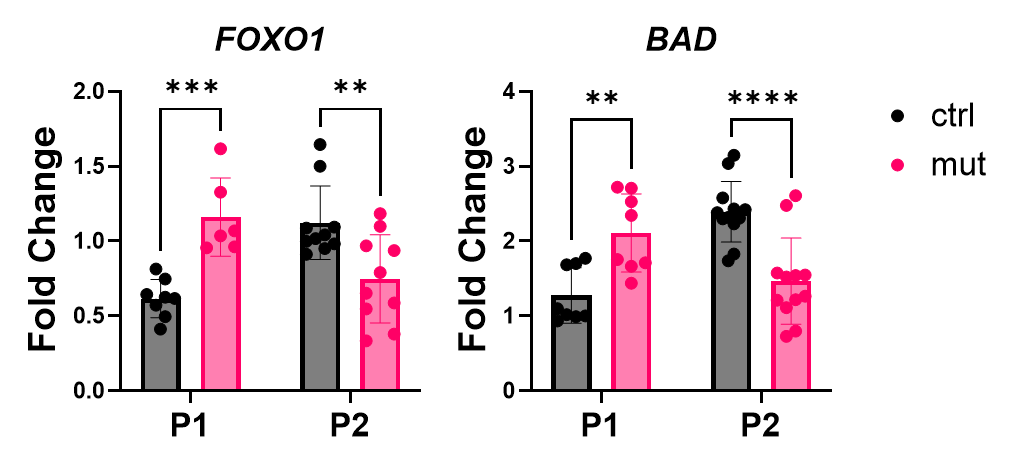


Supplementary Figure 7. Gene expression of apoptosis-regulating factors *FOXO1* and *BAD*. Data are presented as mean ± SD, n=6-12. Statistical analysis was performed using two-way ANOVA followed by Sidak’s multiple comparisons test. **p < 0.01, ***p < 0.001, ****p < 0,0001.

Supplementary Figure 8. Effect of varying concentrations of the BCL2 inhibitor ABT-737 on cell viability after 24-hour treatment. Data are presented as mean ± SD, n=4-6. Statistical analysis was performed using two-way ANOVA followed by Sidak’s multiple comparisons test. **p < 0.01, ***p < 0.001, ****p < 0,0001.


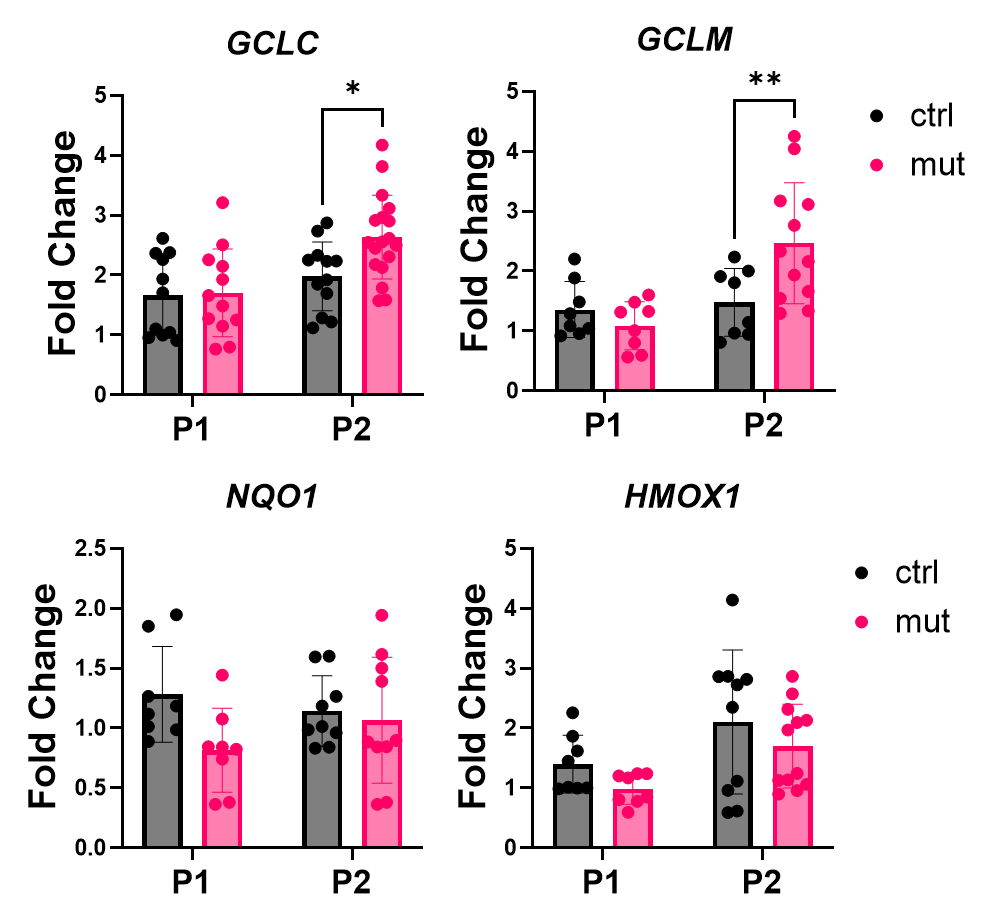


Supplementary Figure 9. Gene expression of NRF2 target genes *GCLC*, *NQO1* and *HMOX1*. Data are presented as mean ± SD, n=6-12. Statistical analysis was performed using two-way ANOVA followed by Sidak’s multiple comparisons test. *p < 0,05, **p < 0.01.


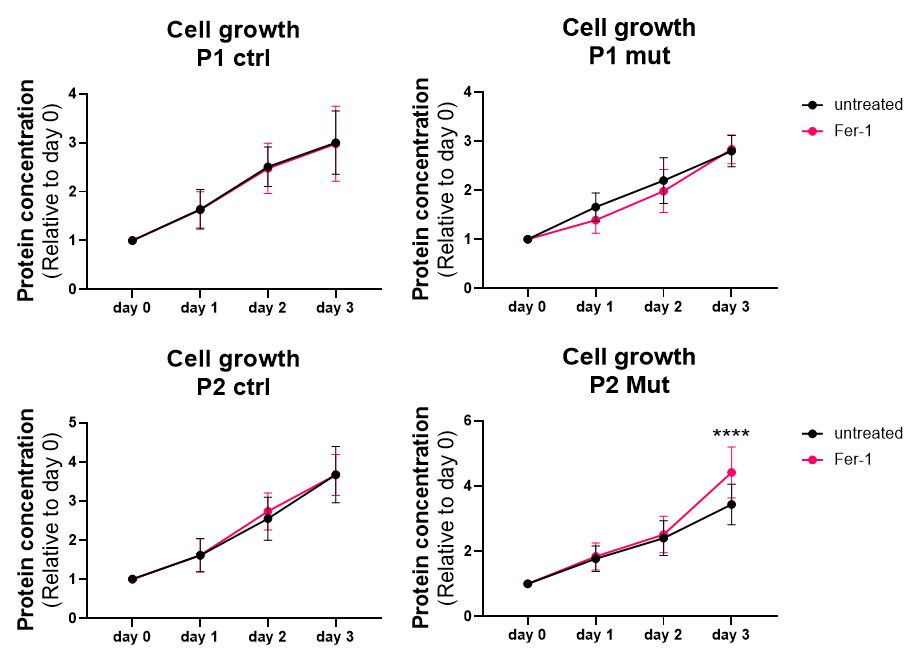


Supplementary Figure 10. Effect of 2 µM ferrostatin-1 (Fer-1) treatment on cell growth over a 3-day period. Data are presented as mean ± SD. Statistical analysis was performed using two-way ANOVA followed by Sidak’s multiple comparisons test. ****p < 0,0001.

Supplementary Table 1. Primers and probes used in the determination of m.3243A>G mutation load.

| Forward primer | CCACCCAAGAACAGGGTTTG |
| --- | --- |
| Reverse primer | GGAATTGAACCTCTGACTGTAAAGTTT |
| 3234A probe | VIC-AGATGGCAGAGCCCGGTA-MGB |
| 3234G probe | FAM-AGATGGCAGGGCCCGGTA-MGB |

Supplementary Table 2. Staining protocols for flow cytometry analysis used in this study.

| **Reagent** | **Concentration** | **Dilution** | **Manufacturer (Product code)** | **Incubation time** |
| --- | --- | --- | --- | --- |
| 2-NBDG | 250 µM | Glucose free RPMI medium | Thermo Fisher Scientific (N13195) | 30 min |
| MitoSOX | 5 µM | Essential 8 medium | Thermo Fisher Scientific (M36008) | 30 min |
| Annexin/PI | 1:100 / 1:100 | Binding Buffer II | Abcam (ab14085) | 5 min |
| BODIPY | 10 µM | Essential 8 medium | Thermo Fisher Scientific (D3861) | 30 min |
| TMRM / MG | 20 nM / 100 nM | Essential 8 medium | Thermo Fisher Scientific (T668) / (M7514) | 15 min |

Supplementary Table 3. Antibodies used in this study.

| **Type** | **Antibody** | **Origin** | **Company (Product code)** | **Working dilution** |
| --- | --- | --- | --- | --- |
| Primary | p62 | Rabbit | Cell Signaling (5114S) | 1:1000 |
| Primary | LC3B | Rabbit | Cell Signaling (2775S) | 1:1000 |
| Primary | Beclin-1 | Rabbit | Cell Signaling (3495S) | 1:1000 |
| Primary | α-tubulin | Mouse | Abcam (ab40742) | 1:4000 |
| Primary | β-actin | Mouse | AssayGenie (CABC004) | 1:4000 |
| Secondary | anti-mouse (HRP) | Goat | Thermo Fisher Scientific (G-21040) | 1:5000 |
| Secondary | anti-rabbit (HRP) | Goat | ImmunoReagents (IMMRIR2219) | 1:5000 |

Supplementary Table 4. Sequences of primers used in this study.

| **Gene** | **Forward** | **Reverse** |
| --- | --- | --- |
| CASP9 | ACAGGCAAGCAGCAAAGTTGTCGA | AGCACCGACATCACCAAATCCTCC |
| CASP8 | CCTCCCTCAAGTTCCTGAGCCT | TTCCCTTTCCATCTCCTCCTTTCT |
| CASP7 | CGGAACAGACAAAGATGCCGAG | AGGCGGCATTTGTATGGTCCTC |
| TP53 | CCTCAGCATCTTATCCGAGTGG | TGGATGGTGGTACAGTCAGAGC |
| BAX | TCACTGAAGCGACTGATGTCCC | ACTCCCGCCACAAAGATGGTC |
| BAK | TTACCGCCATCAGCAGGAACAG | GGAACTCTGAGTCATAGCGTCG |
| BCL2 | ATCGCCCTGTGGATGACTGAGT | GCCAGGAGAAATCAAACAGAGGC |
| BCL2L1 | GCCACTTACCTGAATGACCACC | AACCAGCGGTTGAAGCGTTCCT |
| MCL1 | CCAAGAAAGCTGCATCGAACCAT | CAGCACATTCCTGATGCCACCT |
| BCL2L2 | CAAGGAGATGGAACCACTGGTG | CCGTATAGAGCTGTGAACTCCG |
| BECN1 | CATGGAGAACCTCAGCCGAA | ACAGCGTTTGTAGTTCTGACAC |
| ATG5 | GCAGATGGACAGTTGCACACAC | GAGGTGTTTCCAACATTGGCTCA |
| ATG7 | GCCCAGATTGTCCTAAAGC | AAGCAGCAGACATTTGACAG |
| ALOX5 | GGAGAACCTGTTCATCAACCGC | CAGGTCTTCCTGCCAGTGATTC |
| ALOX15 | ACCTTCCTGCTCGCCTAGTGTT | GGCTACAGAGAATGACGTTGGC |
| TFRC | ATCGGTTGGTGCCACTGAATGG | ACAACAGTGGGCTGGCAGAAAC |
| SCL40A1 | GAGACAAGTCCTGAATCTGTGCC | TTCTTGCAGCAACTGTGTCACAG |
| DR4 | GTGTGGGTTACACCAATGCTTCC | CCTGGTTTGCACTGACATGCTG |
| FAS | GGACCCAGAATACCAAGTGCAG | GTTGCTGGTGAGTGTGCATTCC |
| FOXO1 | CTACGAGTGGATGGTCAAGAGC | CCAGTTCCTTCATTCTGCACACG |
| BAD | CCAACCTCTGGGCAGCACAGC | TTTGCCGCATCTGCGTTGCTGT |
| HMOX1 | TTCAAGCAGCTCTACCGCTC | GAACGCAGTCTTGGCCTCTT |
| NQO1 | TGCAGCGGCTTTGAAGAAGAAAG | TCGGCAGGATACTGAAAGTTCGC |
| GCLC | ATGGAGGTGCAATTAACAGAC | ACTGCATTGCCACCTTTGCA |


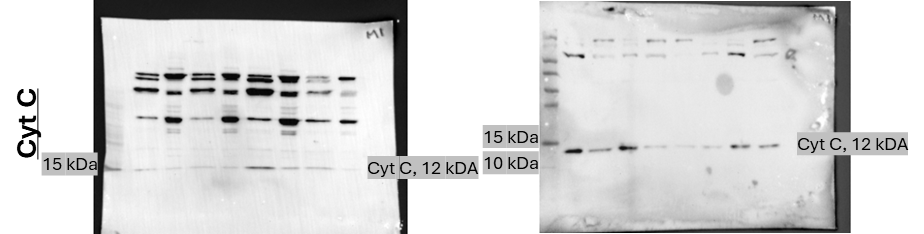


Supplementary Figure 11. Original Western blot images for the detection of Cytochrome C (Cyt C, 12 kDa) corresponding to Figure 3E.


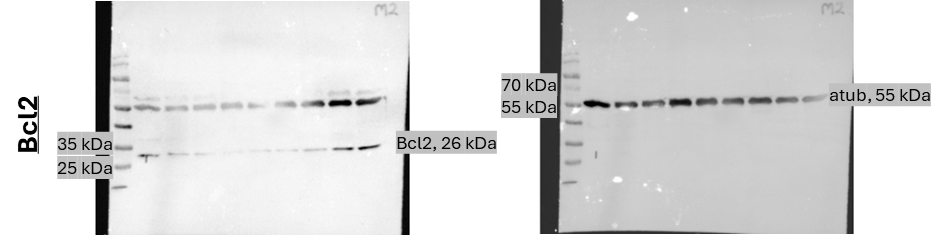


Supplementary Figure 12. Original Western blot images for the detection of Bcl2 (26 kDa) and a-Tubulin (atub, 55 kDa) corresponding to Figure 4C.


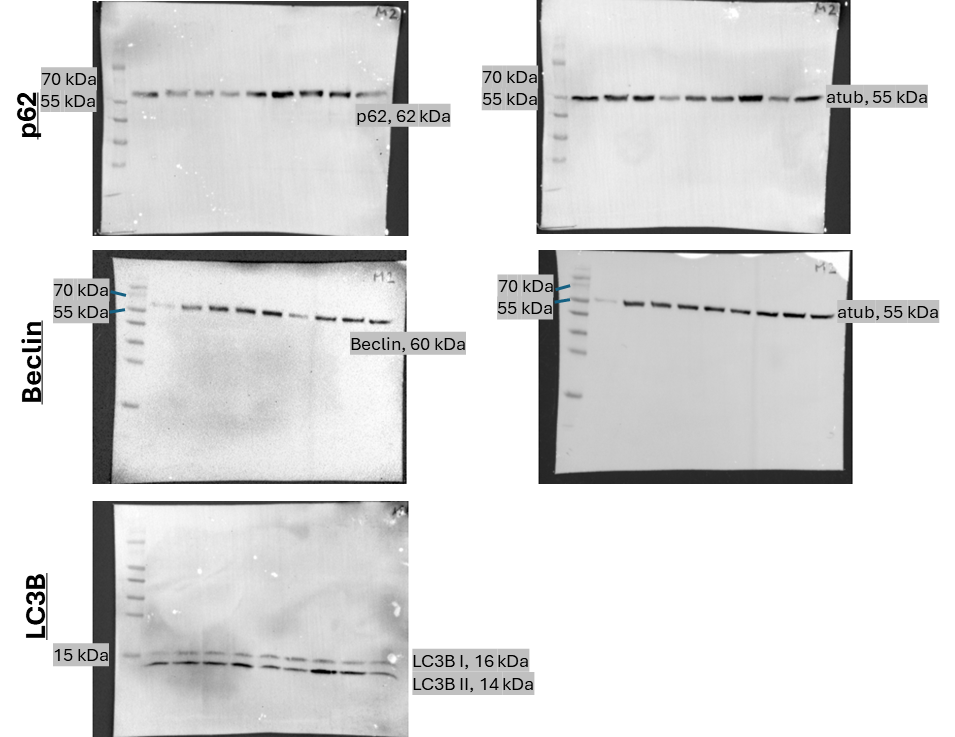


Supplementary Figure 13. Original Western blot images for the detection of p62 (62 kDa), Beclin (60 kDa), LC3B (16 kDa and 14 kDa) and a-Tubulin (atub, 55 kDa) corresponding to Figure 5B.


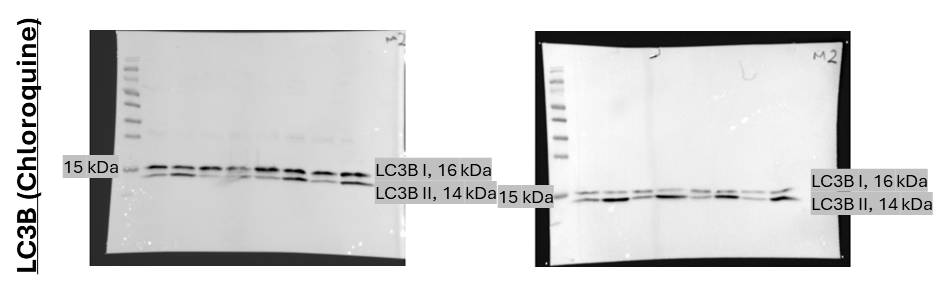


Supplementary Figure 14. Original Western blot images for the detection of LC3B (16 kDa and 14 kDa) corresponding to Figure 5C.
